# Implication of Aging Related Chronic Neuroinflammation on COVID-19 Pandemic

**DOI:** 10.3390/jpm10030102

**Published:** 2020-08-26

**Authors:** Paola Bossù, Elisa Toppi, Valentina Sterbini, Gianfranco Spalletta

**Affiliations:** 1 Experimental Neuropsycho-Biology Lab, Clinical and Behavioral Neurology, IRCCS Fondazione Santa Lucia, Via del Fosso d Fiorano 64, 00143 Rome, Italy; e.toppi@hsantalucia.it (E.T.); v.sterbini@hsantalucia.it (V.S.); 2 Neuropsychiatry Lab, Clinical and Behavioral Neurology, IRCCS Fondazione Santa Lucia, Via Adeatina 306, 00179 Rome, Italy; g.spalletta@hsantalucia.it

**Keywords:** brain inflammation, elderly, SARS-CoV-2, severe COVID-19, COVID-19 neurological and psychiatric manifestations

## Abstract

SARS-CoV-2, the virus responsible for the COVID-19 pandemic, leads to a respiratory syndrome and other manifestations. Most affected people show no or mild symptoms, but the risk of severe disease and death increases in older people. Here, we report a narrative review on selected studies targeting aging-related chronic neuroinflammation in the COVID-19 pandemic. A hyperactivation of the innate immune system with elevated levels of pro-inflammatory cytokines occurs during severe COVID-19, pointing to an important role of the innate immune dysregulation in the disease outcome. Aging is characterized by a general condition of low-grade inflammation, also connected to chronic inflammation of the brain (neuroinflammation), which is involved in frailty syndrome and contributes to several age-associated diseases, including neurodegenerative and neuropsychiatric disorders. Since neuroinflammation can be induced or worsened by the virus infection itself, as well as by stressful conditions like those linked to the recent pandemic, the role of neuroinflammatory mechanisms could be central in a vicious circle leading to an increase in the mortality risk in aged COVID-19 patients. Furthermore, triggered neuroinflammatory pathways and consequent neurodegenerative and neuropsychiatric conditions might be potential long-term complications of COVID-19. In order to provide insights to help clinicians in identifying patients who progress to a more severe case of the disease, this review underlines the potential implications of aging-related neuroinflammation in COVID-19 pandemic.

## 1. Introduction

In December 2019, a novel betacoronavirus has been isolated in China [1]. Apparently arisen from a bat virus adapted to humans via natural recombination or mutation [2], the new virus, named severe acute respiratory syndrome coronavirus-2 (SARS-CoV-2), rapidly spread all over the world, causing an ongoing global health emergency [3,4] with hundreds of thousands of deaths, still rising in number despite measurements of prevention. COVID-19, the highly transmissible disease induced by the new coronavirus, is associated with a range of symptoms, including a mild to moderate respiratory illness in most cases. In aged people and chronically ill patients it is more likely related to a severe condition of pneumonia, which can progress to acute respiratory distress syndrome (ARDS) and multi-organ dysfunctions [5,6]. A growing number of studies observed an increase in COVID-19 severity with age and a higher risk of death in the elderly, as compared to other age groups [7,8,9,10]. In addition, worse outcomes and increased mortality regard men [11] and especially those with multiple age-related diseases, pointing to COVID-19 as an emergent disease of aging, whereas therapeutic approaches addressed to modulate the aging process may be considered to be of some value. Of interest, some patients, especially those with severe COVID-19, suffer from a cytokine storm syndrome and hyperinflammation, an immune overreaction that is mostly deadly to old people [12]. Indeed, the severity of the disease appears to be linked both to the viral infection and the host response, with mortality related to virally driven immune overreactions [13,14,15,16]. Thus, studies addressed to investigate the mechanisms of the malfunctioning anti-viral immune response in people at increased risk of developing severe COVID-19 are of crucial importance to develop efficacious defense strategies. Other coronaviruses able to infect humans have been previously described [17]. Among these, some are associated with mild symptoms resembling those of the common cold (e.g., HKU1, NL63, OC43 and 229E) [18], while two of them, namely the severe acute respiratory syndrome coronavirus (SARS-CoV) and the Middle East respiratory syndrome coronavirus (MERS-CoV), hit the lower respiratory tract and can cause more severe diseases [19,20], likewise the newly identified virus. Of note, the latter two betacoronaviruses share some similarities with the new SARS-CoV-2, especially regarding pathophysiological and immunological features [21], and thus have been used for comparative studies to explore the emerging COVID 19 pathobiology, which is still incompletely decoded. Eventually, similar to what has been reported for other betacoronaviruses, data confirmed that COVID-19 does not affect only the lungs and respiratory system, but older patients with severe infection commonly also have neurologic manifestations [22,23,24]. 

**Figure 1:**
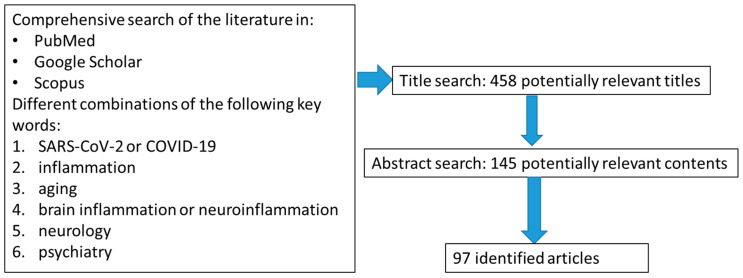
Summary of the consulted and identified scientific articles: selection strategy.

In order to further clarify the causes behind the sharp increase of COVID-19 fatality with age, in this narrative review we explore in general the mechanisms of inflammatory reactions modified by senescence, and focus in particular on age-dependent chronic neuroinflammation and related neurocognitive and neuropsychiatric disorders. Such brain conditions, frequently observed in older people, may relate to COVID-19, possibly being involved in the development of disease neurological/psychiatric manifestations, facilitating SARS-CoV-2-induced respiratory damage and organ failure and acting as a potential hazard for the future health of recovered patients. For these reasons, aging-dependent chronic neuroinflammation represents an interesting topic for discussion for stimulating an early identification and management of high-risk severe cases of COVID-19 and for a better understanding of its pandemic consequences. In particular, as summarized in Figure 1, we have conducted a review of the literature to identify studies investigating the implication of aging-related chronic inflammation and providing evidence or suggestions on the impact of neuroinflammation in the COVID-19 pandemic. We employed commonly used academic engines (PubMed, Google Scholar, Scopus) to search relevant literature through July 2020, and the following key words: “SARS-CoV-2” or “COVID-19”, “inflammation”, “brain inflammation” or “neuroinflammation” have been used in combination with any of the following terms: “aging”, “neurology” and “psychiatry”. The reference list of identified original articles was also hand-searched to obtain additional articles. Studies were considered for inclusion if they were published in English in a peer-reviewed journal; included patients diagnosed with the neuropsychiatric disorders of interest according to International Classification of Diseases (ICD) or Diagnostic and Statistical Manual of mental disorders (DSM) criteria, and included a minimum number of patients to be sufficiently informative. Studies were excluded if there were no data about patients’ neuropsychiatric disorders; sample characteristics were not sufficiently described; and if the study was a case series report of no interest. A total number of 97 studies were considered. Eligibility assessment and study selection were performed by a first author (PB) and independently checked by another author (GS). Overall, the aim of the present review is to obtain a global and synthetic perspective on a topic of emergent interest in the new field of the COVID-19 pandemic and not to describe extensively data of all published studies. Thus, here data of reports describing similar implications might also be represented by individual studies.

## 2. Immune Response against SARS-CoV-2 and Effect of Aging

SARS-CoV-2 is able to enter and infect the cells triggering an immune response in the host that, like other viruses, can be or can not be resolving depending on several factors, including properties of the infecting virus itself, the circumstances of infection and other factors controlled by the host. The factors influencing SARS-CoV-2 pathogenesis and COVID-19 outcome have not yet been fully identified, but it is widely accepted that the increased severity and mortality of the disease are due to not only the viral infection but also the host response. 

Transmission of SARS-CoV-2 occurs mainly via respiratory droplets. After inhalation, the virus is able to gain entry inside host epithelial cells and pneumocytes through the binding of its spike (S) glycoprotein to the angiotensin-converting enzyme 2 (ACE2), in concert with the action of host proteases, principally transmembrane serine protease 2 (TMPRSS2) [25]. Following host cell infection, SARS-CoV-2 triggers both innate and specific immune response, but the precise immune pathways involved in the activation of the anti-virus host defense mechanisms remain to be elucidated. It has been hypothesized that host-infected cells release danger signal molecules after undergoing pyroptosis, or can be directly recognized by immune cells. These processes may trigger inflammasome activation with consequent involvement of IL-1β pathways, and lead to the release of pro-inflammatory cytokines and chemokines, which in turn attract monocytes, macrophages and T cells to the site of infection, promoting further inflammation in the subject at risk, or leading to a resolving immune response in healthy individuals [21]. In agreement, the levels of circulating IL-1β have been found elevated in SARS-CoV-2-infected patients and high levels of other cytokines, such as TNF, IL-6, IL-8, G-CSF and GM-CSF, as well as chemokines including MCP1, IP10 and MIP1α, have been observed in critically ill patients [12,26]. These results suggest that the COVID-19 pathologic process may involve a dysfunctional immune response characterized by an exaggerated pro-inflammatory cytokine response, evoking a cytokine storm [12], which can compromise tissue integrity and function and contribute to acute respiratory distress syndrome and multiple organ dysfunction syndrome [27,28]. Indeed, inflammation appears to be more conspicuous in severe than in moderate COVID-19 cases, and in deceased victims more than in recovered patients, suggesting that inflammatory processes play a critical role in disease progression and mortality after SARS-CoV-2 infection [6,13]. Furthermore, a poor COVID-19 outcome has been associated with age-dependent defects in T-cell and B-cell function and with an excess production of type 2 cytokines, which could lead to a deficiency in control of viral replication and a more prolonged pro-inflammatory response outcome [7,29]. In severe COVID-19 patients, an aberrant mononuclear inflammatory infiltrate dominated by lymphocytes has been found in the lungs [30] and in peripheral blood decreased counts of T cells characterized by reduced functional diversity and exhaustion have been also observed [31,32]. Taken together, these results suggest that T cells may have a role in controlling the disease, while in severe disease conditions there is an imbalanced host response characterized by increased inflammation and decreased T cell functions [31].

A large number of evidence reports that frail older adults have a senescent immune system characterized by inflammaging, a complex dynamic of inter-related processes leading to a chronic condition of elevated inflammation, which contributes to the pathogenesis of age-related diseases. [33]. Inflammaging, likely due to the prolonged or chronic antigen stimulation occurring during the lifetime and insults that accumulate over time such as oxidative stress, induces pro-inflammatory traits, increased risk of autoimmunity, and diminished acquired immunity against infection. Both innate and adaptive immune functions are affected in inflammaging. Specifically, monocytes from older individuals exhibit altered phenotype and function, including an increase in CD16 expressing subsets (nonclassical and intermediate monocytes) that produce proinflammatory cytokines [34], and metabolic reprogramming accompanied by signs of increased oxidative stress and DNA damage [35]. Proinflammatory cytokines may attract and activate innate immune cells, including neutrophils and monocytes, which release oxidizing molecules that in turn can damage tissues, concurring to establish the sustained level of inflammation and ultimately resulting in physiopathology of frailty and increased severity of viral infections in elderly individuals [36,37]. These immune alterations may have implications for the aging-related progression of COVID-19, as supported by emerging studies on patients infected by SARS-CoV-2, which describe modifications regarding monocytes (including inflammatory CD16+ monocytes) and unbalances of cellular and systemic cytokine levels in association with COVID-19 progression [15,38,39].

Along with the altered innate immune response, adaptive immune system deviations also characterize the advanced age and a disturbed balance between inflammaging and immune senescence is observed, including altered T cell functions and a reduced number or abrogation of naïve T cells depending on age-dependent thymus atrophy [40]. In addition, a senescent state of accumulated and exhausted lymphocytes that secrete preferentially pro-inflammatory cytokines has been described in aged subjects [41,42]. Such a series of events leading to an increased susceptibility to develop chronic inflammatory diseases results in a decreased ability to fight viral infections, especially in frail patients [43]. Altogether, these preexistent conditions may worsen COVID-19 outcomes and concur to favor hyper inflammation and a deadly cytokine storm, affecting older people during the ongoing pandemic. 

Linked to inflammaging and increased oxidative stress processes, elderly people commonly show age-related vascular inflammation with an increased release of reactive oxygen species, which contribute to endothelial dysfunction and predisposition to both microvascular and macrovascular diseases [44]. Of interest, preexisting cardiovascular and cerebrovascular diseases are risk factors for severe COVID-19 outcome and older patients with risk factors are more likely to develop them [45,46]. In addition, patients with COVID-19 infection may be at a greater risk of ischemic stroke than patients with influenza infection [47], strengthening the link between the COVID-19-associated inflammatory response and vascular disturbances. Another important mechanism that could influence COVID-19 progression by means of immune response regulation in the elderly is trained innate memory. This relatively new concept of immunology regards the ability of the innate immune system to undergo nonspecific long-term responses able to provide resistance by modifying the reactivity to sequential pathogen challenges by means of epigenetic and metabolic reprogramming of innate immune cells [48]. Under certain circumstances, trained innate memory mechanisms can boost the innate immune responses to control viral replication, thus the induction of innate immune reprogramming before infection by using, for instance whole-microorganism vaccines, has been proposed as a potential approach for boosting antiviral responses and reducing susceptibility to and severity of SARS-CoV-2 [49]. However, individuals are exposed to a multiplicity of different threatening and pathogenic agents during their life, and thus a large heterogeneity in innate memory response is expected. It is also possible to speculate that older people, who have had multiple chances to encounter pathogens during their life, much more than younger individuals, possess trained innate immune cells reprogrammed to facilitate inflammation in response to acute inflammatory stimuli, like SARS-CoV-2 infection, favoring a hyperinflammatory loop and COVID-19 severe complications. In keeping, this concept has been previously applied to other aging-associated pathological conditions, where under predisposing situations, innate memory may endorse human diseases characterized by excessive inflammation, even causing a neuroinflammatory cycle in neuropathological conditions, including Alzheimer’s disease [50,51,52]. Overall, the innate memory-based approaches to modulate the host defense against SARS-CoV-2 might contribute to reducing the spread of the infection and deserve to be pursued. However, more conventional approaches addressed to prevent hyper-inflammatory host responses may exert some beneficial effects. Consistent with this, clinical trials with inhibitors of IL-6 or IL-1 pathways are underway and show preliminary but encouraging results [53,54], confirming the central role of overreacting inflammatory mechanisms and cytokine storms in exacerbating COVID-19 severity. 

## 3. Neuroinflammation, Neurological and Psychiatric Aspects in COVID-19

The extensive and reciprocal relationships existent between the immune system and the central nervous system (CNS) have gained great attention in the last decade. Specifically, peripheral immune responses and activation of innate immune mechanisms leading to pro-inflammatory cytokine up-regulation can act on the brain and induce behavioral alterations that are reminiscent of sickness, including depressed mood, reduced locomotor activity and social exploration, loss of appetite, sleepiness and hyperalgesia. Growing evidence shows that inflammatory processes profoundly affect brain functions leading to the development of symptoms of depression in vulnerable individuals [55] and are involved in neurological illnesses [56], including aging-dependent neurodegenerative diseases, such as Alzheimer’s disease leading to dementia [57]. 

The propagation of inflammation from the periphery to the brain occurs by means of different pathways, whose activation can exacerbate immunologic contributions to neuropsychiatric disorders [58]. During peripheral inflammatory response, trafficking of blood-borne immune cells into the brain may also occur, contributing to the cerebral inflammatory milieu. In more detail, soluble pro-inflammatory mediators may diffuse from the circumventricular organs outside the blood–brain barrier (BBB), can activate sensory afferents of cranial nerves, interact with a transport system at the BBB, or can also be directly secreted by the cells that constitute the BBB itself. Thus, the BBB function is crucial to allow the correct immune-to-brain communication. Of interest, aging, as well as exuberant inflammatory responses in the periphery, can promote some disturbance in the BBB, thus further enabling the trafficking of immune cells to the brain and supporting potential neuropathological processes [59,60,61]. When the recruitment of peripheral immune cells into the brain occurs and the CNS resident immune cells (microglia) undergo a sustained activation status characterized by an increased release of pro-inflammatory cytokines, a condition of chronic neuroinflammation arises. In this context, advanced age and comorbid diseases with an inflammatory component have a powerful effect in enhancing neuroinflammatory processes and susceptibility to neurodegenerative diseases [62]. However, the degree of neuroinflammation and its positive or negative consequences appears to depend on the context, duration, and course of the primary stimulus or insult. Thus, to decipher the individual situations and specific settings in which the neuroinflammation is occurring is of great importance for managing brain damages [63]. Accordingly, individualized therapeutic strategies targeting neuroinflammation under the framework of precision medicine are considered to be likely efficacious in controlling the pathogenic pathways leading to neurodegeneration [64].

Given that COVID-19 patients are generally older and with comorbid conditions when severely affected as compared with those with a moderate disease, they often suffer from chronic inflammation, microvascular inflammation, vasculature damages and endothelial dysfunctions [65]. Therefore, the possibility that SARS-CoV-2 is associated with some level of BBB perturbation and neuroinflammation is highly conceivable. The preexisting inflammatory component and increased vulnerability to neuroinflammation could be further strengthened by the possible neuro-invasive ability of SARS-CoV-2 [66], and post infection immune-mediated processes [67], which in turn can lead to brain hyperproduction of cytokines. Previous SARS-CoV-1 studies [68,69] and a few COVID-19 patients showing inflammatory changes in the CSF [70] sustain the potential of such a sequence of events. That an association between the virus and the brain exists is confirmed by clinical observations reporting that about one third of severe COVID-19 patients present neurological symptoms including headache, dizziness, altered consciousness, ischemic and hemorrhagic strokes [23]. As reported in a meta-analysis study, among the psychiatric presentations of COVID-19, delirium is probably a significant clinical problem in the acute stage and neurocognitive impairment might be a feature in patients with severe disease. In the longer term, there is the possibility of depression, anxiety, fatigue, post-traumatic stress disorder and rarer neuropsychiatric syndromes [71]. Headache and anosmia are the most common neurological manifestations of SARS-CoV-2 infection [72], but meningitis, encephalitis, hemorrhagic necrotizing encephalopathy, Guillain-Barré syndrome, cerebrovascular diseases and ischemic stroke have been further described in association with COVID-19 in some patients and in multiple clinical contexts [73,74,75,76,77,78,79]. Of interest, patients who show neurological symptoms included cases with or without pre-existing neurological disorders [79]. In addition, COVID-19 patients with a history of neurologic disease result at an increased risk of becoming critically ill or dying, as compared to patients without COVID-19 [80], and it has also been suggested that the neuro-invasive potential of SARS-CoV2 and the consequent brain lesions may be responsible for the respiratory failure observed in severe COVID-19 patients [81]. Within such a vicious circle with detrimental effects on COVID-19 progression, neuroinflammation may play a crucial role. Furthermore, a cross-specialty surveillance study of acute neurological and psychiatric complications of COVID-19 shows that ischemic stroke is common especially in older patients, and a large group of patients shows altered mental status, reflecting both neurological and psychiatric diagnoses, such as encephalitis and psychosis. Such altered mental status is identified across all age groups, with many younger patients having this presentation [82].

Regardless the current limited knowledge of SARS-CoV-2 neuro-invasive mechanisms and effects on brain integrity, a growing number of studies have been addressed to the impact of preexisting neurological disturbances on the disease progression, as well as to the neurological consequences of COVID-19 [72,83,84,85,86], and an impact of the virus on long term neurodegenerative or neuropsychiatric disturbances has been hypothesized [87,88]. In particular, a damaging immune overreaction, or an acceleration or aggravation of pre-existing cognitive deficits, or even a de novo induction of a neurodegenerative disease have been hypothesized to determine a higher risk of developing cognitive decline in SARS-CoV-2 infected patients [89] 

In relation to the neuropsychiatric aspects of COVID-19, other than the increase in mental health conditions in both the short and long term as consequences of the pandemic, it is also conceivable that prolonged stressful situations, like the fear of infection and social distancing, may worsen neuroinflammation in frail subjects, affecting CNS homeostasis and thus acting as an accelerator of COVID-19 progression. Chronic stressors are in fact a major trigger for chronic low-grade inflammation and persistent neuroinflammatory processes, leading to a wide variety of chronic diseases [90]. In line with this view, a marked cortisol stress response has been observed in severe COVID-19 patients who show high serum total cortisol concentrations associated with increased mortality [91]. 

Altogether, the neurological disorders associated with COVID-19 are variable and apparently do not follow a specific pattern. Consistently, the neurological damages caused by SARS-CoV-2 as well as other coronaviruses, are multifaceted, implying direct infection pathways (blood circulation pathways and neuronal pathways), hypoxia, immune injury, ACE2, and other mechanisms [84]. A better knowledge of the clinic-radiological manifestations of COVID-19 could be helpful to find the best care for patients and for understanding of the disease [92]. However, more detailed studies addressed to investigate early neuropsychiatric symptoms and infection-related neurological complications in COVID-19 patients are urgently required. Overall, future efforts and systematic strategies should be employed in order to understand whether the described neurological disorders are caused by the virus infection or rather are a consequence of severe systemic disease on underlying neuropathological conditions. In any case, the pathogenic mechanisms involved in the emergence of COVID-19-linked neurological disturbances need to be thoroughly assessed in the future, including the evaluation of neuroinflammatory processes, which could be crucially useful for the early identification and management of high-risk severe cases. Currently, optimized positron emission tomography (PET) radiotracers are under development to track neuroinflammation [93]. PET imaging is certainly a powerful non-invasive technique to monitor the spatiotemporal dynamics of various immune cells in living subjects. It is currently used in clinical research to explore neuroinflammation in neurological diseases and is promising to become a tool with use in routine clinical contexts. The identification of a specific cytokine profile in older subjects able to predict COVID-19 progression in a routine clinical settings is linked to the definition of a chronic low-grade inflammation profile, a topic still under thorough research. Specifically, in aging conditions, pro-inflammatory factors including IL-6, IL-8, IL-2, IFN-γ, and TNF-α are at levels higher than baseline [94,95], even though they are many-fold lower than those measured in acute inflammation. Overall, a balance between pro- and anti-inflammatory factors shapes the inflammatory status of elderly patients’ plasma and molecules involved in inflammaging appear to work within a biological context characterized by complex and still not fully clarified molecular interactions and pathways. Nonetheless, despite both frail and healthy centenarians often having plasma levels of pro-inflammatory mediators that are higher than those of young individuals, some molecules are elevated exclusively in centenarians. It is this case, for instance, of CCL5 (RANTES), which is higher in centenarians’ blood, suggesting that T lymphocyte recruitment towards sites of inflammation may have protective implications [96]. Interestingly, in line with the last observation and with the reported decreased T cell functions in severe COVID-19 [26], the CCL5 molecule is elevated from the early stages post-infection in the plasma of COVID-19 patients with mild but not severe disease [97], promoting its candidacy among the potential prognostic markers useful to predict SARS-CoV-2 infection outcomes.

Overall, the possibility in the future to measure neuroinflammation in each single patient by using PET imaging or by detecting inflammatory profiles in CSF or blood, or even combining them [64], would facilitate the identification of patients who could progress to severe disease, allowing individualized therapies and tailored treatments for COVID-19.

## 4. Conclusions

**Figure 2:**
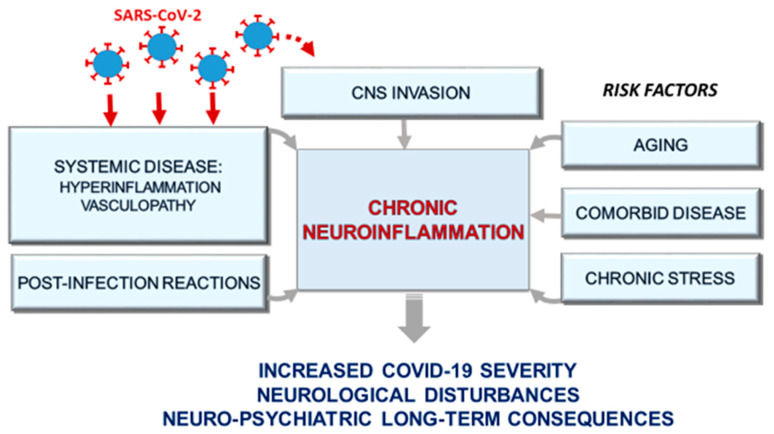
Potential impact of chronic neuroinflammation on COVID-19 severity: a detailed explanation of the drawing is reported in the text. CNS: Central Nervous System.

Accumulating evidence indicates that SARS-CoV-2 infection can affect multiple organs beyond the respiratory system and it is often associated with neuropsychiatric manifestations. Even though aging and comorbid conditions appear to be involved in severe COVID-19 and its related neuropsychiatric alterations, the underlying mechanisms that characterize SARS-CoV-2 direct or indirect effects on the CNS are still unknown. Neuroinflammation may be a central phenomenon in this context, as depicted in Figure 2. In fact, the same conditions that may play as risk factors for severe COVID-19, namely aging and comorbid chronic diseases including diabetes, cardiovascular disease and hypertension, as well as chronic stress, are associated with upregulation of inflammation and presumably with predisposition to neuroinflammatory processes. In addition, neuroinflammation could be promoted as part of the sustained anti-viral immune response arising in the periphery (e.g., cytokine storm) and possibly in the brain following viral neuro-infection. Furthermore, since neurodegenerative processes evolve over decades, SARS-CoV-2 infection could trigger a sequela of immune-mediated events able to promote chronic neuroinflammation, which in turn can lead to neuronal damage and degeneration in the long term, possibly producing chronic neurological consequences from this pandemic. Thus, we propose to implement non-invasive evaluation of neuropsychiatric functions including brain inflammatory status, which can in future direct COVID-19 prognosis, as neuroinflammation may be a missing link in a vicious circle of pandemic-devastating effects on aged generation.
